# microRNA-211-5p predicts the progression of postmenopausal osteoporosis and attenuates osteogenesis by targeting dual specific phosphatase 6

**DOI:** 10.1080/21655979.2021.2017626

**Published:** 2022-02-21

**Authors:** Huan Wang, Xiaoyan Shi, Zhenye Guo, Feng Zhao, Weifu He, Mingming Kang, Zhi Lv

**Affiliations:** a Department of Orthopaedics, Second Hospital of Shanxi Medical University, Taiyuan, Shanxi, China; b Department of Orthopaedics, West Hospital of Second Hospital of Shanxi Medical University, Taiyuan, Shanxi, China

**Keywords:** Osteoporosis, osteogenic differentiation, miR-211-5p, DUSP6, ERK

## Abstract

Postmenopausal osteoporosis (PMOP) is known as one of the prevalent diseases among middle-aged and elderly women. This paper revolves around the alteration of miR-211-5p in PMOP patients and its function in osteogenic differentiation. Quantitative real-time polymerase chain reaction (qRT-PCR) was implemented to check the miR-211-5p level in the plasma of PMOP patients. Knockdown and overexpression experiments were done to verify the influence of miR-211-5p on human-derived mesenchymal stem cell (hMSC) osteogenic differentiation and osteogenesis. The alkaline phosphatase (ALP) assay kit was taken to test ALP activity. Alizarin red staining monitored osteogenic differentiation, while oil red O staining examined adipogenesis. Western blot confirmed the profiles of osteoclastogenesis-concerned factors (TRAP, NFAT2, c-FOS, Runx2, OCN, CTSK), dual specific phosphatase 6 (DUSP6), ERK, SMAD, and β-catenin. Dual-luciferase reporter and RNA immunoprecipitation assays were implemented to identify the association between miR-211-5p and DUSP6. Our data displayed that miR-211-5p was down-regulated in the PMOP patients’ plasma (in contrast with the healthy controls), and it was positively correlated with Vit-D and BMD levels. miR-211-5p overexpression vigorously facilitated hMSC osteogenic differentiation, while miR-211-5p inhibition contributed to the opposite situation. miR-211-5p initiated the ERK/SMAD/β-catenin pathway and repressed DUSP6’s expression. Overexpression of DUSP6 counteracted the miR-211-5p-mediated function to a great extent and inactivated ERK/SMAD/β-catenin, whereas enhancing ERK phosphorylation weakened the DUSP6 overexpression-induced function. Consequently, this research unveiled that miR-211-5p promotes osteogenic differentiation by interfering with the DUSP6-mediated ERK/SMAD/β-catenin pathway.

## 1. Introduction

Postmenopausal osteoporosis (PMOP) is a metabolic disease stemming from a decline in the ovarian endocrine function following menopause, which contributes to a reduction in the estrogen level and thus makes osteoclast bone resorption bigger than osteoblast bone formation. The patients are prone to fracture due to a decrease in bone mass and an increase in osteopsathyrosis, and fracture may be followed by bone deformation, pain, other complications, and even death [1]. With no specific clinical manifestations, osteoporosis (OP) will not be taken into account until a minor trauma incurs a fracture [2]. Therefore, OP prevention is the top priority in clinical practice.

MicroRNAs (miRNAs), small non-coding RNAs, suppress messenger RNA (mRNA) translation or expedite mRNA degradation to modulate gene expression at the post-transcriptional level, hence regulating umpteen physiological and pathological processes like cancers, cardiovascular diseases, and metabolic diseases [3–5]. What’s more, the functions of miRNAs in OP have been substantiated by several studies [6]. For instance, by engineering a PMOP model in rats through bilateral oophorectomy (OVX), some researchers have discovered that miR-133a knockdown alters the serum levels of factors pertaining to osteoclast formation, augments the lumbar bone mineral density (BMD), and changes bone morphology [7]. miR-19a-3p cramps HDAC4’s expression to step up human-derived mesenchymal stem cell (hMSC) osteogenic differentiation, thereby mitigating OP [8]. All these findings have unraveled that different miRNAs exert positive or negative regulatory functions in OP.

Dual specific phosphatase 6 (DUSP6), a pivotal gene of OP [9], is a member of the bi-specific protein phosphatase subfamily [10]. So far, researches on DUSP6 have primarily concentrated on tumors and inflammatory diseases. For instance, inhibition of DUSP6 modulates ERK signal response genes to boost ovarian cancer cells’ sensitivity to chemotherapy drugs [11]. (E/Z)-BCI hydrochloride (BCI), a small molecular inhibitor of DUSP6, attenuates lipopolysaccharide (LPS)-elicited inflammatory mediators and ROS generation in macrophages by initiating Nrf2 and inactivating NF-κB. These anti-inflammatory functions suggest that BCI can be adopted as a therapeutic agent for blocking inflammatory diseases [12]. On the other hand, DUSP6 mediates glycolysis involving T cell receptors and dampens T_FH_ cell differentiation through IL-21 production inhibition [13]. Notwithstanding, we are still in the dark about the certain function and corresponding molecular mechanism of DUSP6 in the context of OP.

As evidenced by a multitude of studies, ERK [14], SMAD [15], and β-catenin [16,17] all participate in OP, and their activation enhances osteogenic differentiation. Of note, uncarboxylated osteocalcin, a hormone derived from osteoblasts, has been uncovered to boost the osteoblastic differentiation of mouse bone marrow-derived mesenchymal stem cells (MSCs) via ERK/SMAD/β-catenin pathway activation [18]. Nevertheless, the function of the ERK/SMAD/β-catenin pathway in hMSCs remains elusive, and whether it is regulated by miR-211-5p or DUSP6 has not been disclosed.

Predicated on the above studies, we posit that miR-211-5p makes a potential role in OP progression. We found that miR-211-5p was down-regulated in the PMOP plasma compared with healthy controls, and miR-211-5p overexpression vigorously facilitated hMSC osteogenic differentiation. In addition, miR-211-5p promoted ERK/SMAD/β-catenin pathway. DUSP6, as revealed by bioinformatic analysis, was a potential target of miR-211-5p. Therefore, we guessed that miR-211-5p gets involved in OP by targeting UDSP6. In conclusion, this paper aims to reveal a novel miR-211-5p-DUSP6-ERK/SMAD/β-catenin axis in OP development, which provides a new reference for OP diagnosis and treatment.

## 2. Materials and Methods

### 2.1 Clinical specimen collection

Here, 95 postmenopausal women at the age of 58 to 68 were selected from the Second Hospital of Shanxi Medical University. Among them, 75 were patients suffering from OP, and 20 were adopted as healthy controls. OP was diagnosed if the femoral neck and/or lumbar T-score was less than or equal to −2.5, with a T-score of −1 or above considered normal. Postmenopausal women were enrolled if they had not had a period for at least 12 months. Patients with metabolic diseases, cardiovascular diseases, malignancies, or other severe diseases over the past five years were ruled out of the study. Also, subjects were excluded if they had been treated with glucocorticoids, estrogens, thyroid hormones, parathyroid hormones, fluoride, bisphosphonates, calcitonin, thiazide diuretics, barbiturates, or antiepileptic drugs [19]. This study had received the imprimatur from the Medical Ethics Committee of Second Hospital of Shanxi Medical University. All participants had signed the informed consent. The morning fasting peripheral venous blood (4–8 mL) of all subjects was harvested, put into a K2-EDTA-coated tube (BD, NJ, USA), and stored at 4°C. Within 24 hours, the blood samples were centrifuged at 1500 RPM for 15 min at 4°C and then at 14,000 RPM for another 15 min at 4°C. Subsequent to centrifugation, the supernatant (plasma) was administered equally to RNase/DNase-free tubes and stored at −80°C in preparation for the following procedures.

### 2.2 Cell culture

hMSCs (HUXMA-01001) were supplied by Cyagen Biosciences (Suzhou, China). This cell line was characterized to be CD73-, CD90-, and CD105-positive (≥ 95%), and CD11b-, CD19-, CD45-, CD34-, and CD HLA-DR-negative (≤ 5%) [20]. The cells were cultured with an oriCell^TM^ human Mesenchymal Stem Cell growth medium (HUXMA-90011, Cyagen Biosciences) incorporating 10% fetal bovine serum (FBS), 1% glutamine, and 1% penicillin, and kept in an incubator at 37°C with 5% CO_2_. Next, the cells were trypsinized employing 0.25% trypsin-EDTA (Gibco; Thermo Fisher Scientific, Inc.). For inducing osteogenic differentiation, the hMSCs were seeded in 60-mm dishes, dealt with 50 mM ascorbic acid, 10 mM β-glycerophosphate, and 100 nM dexamethasone (Sigma-Aldrich, St. Louis, MO) for 20 days (the medium was refreshed every 3 days). For inducing adipogenic differentiation, the hMSCs were seeded in 60-mm dishes, and the culture medium encompassed human recombinant-insulin (SingleQuots™), L-glutamine, MCGS, dexamethasone, indomethacin, IBMX (3-isobutyl-l-methyl-xanthine), and GA-1000 (Lonza, Walkersville, MD, USA).

### 2.3 Cell transfection

hMSCs were transfected along with miR-211-5p mimics to get a miR-211-5p overexpression model established. On the contrary, the miR-211-5p inhibitor was transfected into hMSCs to engineer a miR-211-5p knockdown model. Meanwhile, hMSCs were transfected along with pcDNA-DUSP6 and pcDNA-vector for the construction of a DUSP6 overexpression model. The cell transfection procedure was performed according to a previous study [21]. The ERK Agonist PAF-C16 (Santa Cruz Biotechnology Inc., 4 μM) was utilized to activate the ERK pathway. Under the same conditions of cell culture, the blank group received no treatment.

### 2.4 Quantitative real-time polymerase chain reaction (qRT-PCR)

As per miR-211-5p and DUSP6 mRNA sequences in Genebank, primers were designed with the assistance of the Primer3.0 software and synthesized by Sangon Biotechnology Co., Ltd (Shanghai). TRIzol was taken to separate total cellular RNA, with the RNA purity and concentration determined using an ultraviolet spectrophotometer. Reverse transcription was implemented to synthesize the first strand of cDNA. With the strand as a template, the mRNA fragments were amplified employing the ABI7300 fluorescent quantitative PCR instrument. GAPDH was regarded as the internal parameter of DUSP6, while U6 was adopted as that of miR-211-5p. The relative profile of the target gene was calculated using the 2^−∆∆CT^ method [22]. The primer sequences of each gene are detailed in Table 1.

**Table 1.** The primer sequence of each gene

Gene Primer sequence (5ʹ→3ʹ)
miR-211-5p F: ACACTCCAGCTGGGCAAGTAGCATCAACTA R: TGGTGTCGTGGAGTCG
DUSP6 F: GAACTGTGGTGTCTTGGTACATT R: GTTCATCGACAGATTGAGCTTCT
GAPDH F: CTCCTCCTGTTCGACAGTCAGC R: CCCAATACGACCAAATCCGTT U6 F: CTCGCTTCGGCAGCACA R: AACGCTTCACGAATTTGCGT

### 2.5 Plasma 25 (OH) D concentration determination

The blood samples harvested from 75 PMOP patients and 20 healthy controls were examined. On the basis of prior studies [23], the plasma concentration of 25 (OH) D [coefficient of variation (CV) was 3.2%] was determined through enzyme-linked immunosorbent assay (ELISA) (Immunodiagnostic Systems Inc., Fountain Hills, AZ). This assay was sensitive to the concentration of 25 (OH) D at 2.0 ng/mL.

### 2.6 BMD determination

A Hologic 4500A dual-energy X-ray absorptiometry (DXA) scanner (Hologic Inc., Bedford, MA) was adopted to check the BMD (g/cm^2^) level of the lumbar spine (L1-4). The instrument disclosed that the coefficient of variation (CV) of the spine was 0.9%.

### 2.7 Alkaline phosphatase (ALP) activity

After being inoculated into 6-well plates (2 × 10^6^ cells/well), hMSCs steadily transfected were flushed with PBS, dealt with 0.25% trypsin (Thermo Fisher HyClone, Utah, USA) for 15–30 min, and centrifuged at 14,000 RPM for 15 min. The ALP assay kit (Merck, Shanghai, China) was utilized to determine ALP activity in the cell supernatant as instructed by the manufacturer [24].

### 2.8 Western blot (WB)

hMSCs were dealt with different factors, and then the culture medium was discarded. The protein lysate (Roche) was administered to isolate the total protein. Afterward, 50 g of the total protein was loaded onto 12% polyacrylamide gel for 2 hours’ electrophoresis at 100 V and then electrically moved onto polyvinylidene fluoride membranes. After being sealed with 5% skimmed milk at room temperature for an hour and flushed with TBST three times (10 min each time), the membranes were incubated overnight at 4°C along with the following primary antibodies: Anti-TRAP antibody (1:5000, ab52750), Anti-NFAT2 antibody (1:1000, ab2796), Anti-c-FOS antibody (1:1000, ab222699), Anti-Runx2 antibody (1:1000, ab23981), Anti-OCN antibody (1:1000, ab93876), Anti-CTSK antibody (1:1000, ab19027), Anti-p-Erk1 antibody (1:1000, ab131438), Anti-Erk1 antibody (1:1000, ab32537), Anti-p-Smad3 antibody (1:1000, ab74062), Anti-Smad3 antibody (1:1000, ab40854), Anti-β-catenin antibody (1:5000, ab32572), Anti-DUSP6 antibody (1:1000, ab76310), and Anti-GAPDH antibody (1:2500, ab9485). TBST was taken to rinse the membranes, which were next incubated along with the horseradish peroxidase (HRP)-labeled anti-rabbit secondary antibody (1:2500, ab6721) at RT for an hour. TBST was applied to rinse the membranes three times, 10 min each. At last, WB reagent (Invitrogen) was utilized for color development, and Image J was adopted to evaluate the gray value of each protein. All antibodies employed in this study were ordered from Abcam (MA, USA) [25].

### 2.9 Alizarin red staining

Following osteogenic differentiation induction, hMSCs were flushed in PBS 3 times and immobilized in 10% formalin for 30 min. Then, the cells were rewashed with PBS and maintained for 3 hours with alizarin red solution at RT. After being washed twice with distilled water, the cells were photographed [26].

### 2.10 Oil red O staining

Subsequent to adipogenic differentiation induction, hMSCs (1 × 10^5^ cells/mL) were dyed employing oil red O and flushed twice in PBS. Then, the cells were immobilized with 10% formalin at 37°C for 10 min, stained adopting filtered oil red O solution at 37°C for an hour, and monitored with a Leica Microsystem fluorescence microscope (DFC300 FX; Leica Microsystems GmbH, Wetzlar, Germany) [27].

### 2.11 Statistical analysis

The SPSS16.0 software (SPSS Inc., Chicago, IL, USA) was introduced for statistical analysis, with the outcomes presented as mean ± SD (x ± s). The *t-*test was taken to compare two groups, while one-way ANOVA with Tukey post hoc test was applied for comparison among multiple groups. Pearson correlation analysis was utilized to assess the correlation among miR-211-5p, vitamin D (Vit-D), and BMD. *P* < 0.05 was regarded as statistically meaningful.

## 3. Results

Aiming at exploring the role of miR-211-5p in OP development, we first detected miR-211-5p level in the plasma samples of PMOP patients and heathy donors. miR-211-5p was downregulated in PMOP patients, we guessed that miR-211-5p has effects on osteogenic differentiation. Thus, we performed gain-and loss-of functional assays of miR-211-5p and DUSP6 on hMSCs for verifying the effects of the miR-211-5p-DUSP6 axis in osteogenic differentiation.

### 3.1 miR-211-5p was knocked down in the plasma of PMOP patients and positively correlated with the plasma levels of Vit-D and BMD

**Figure 1.**
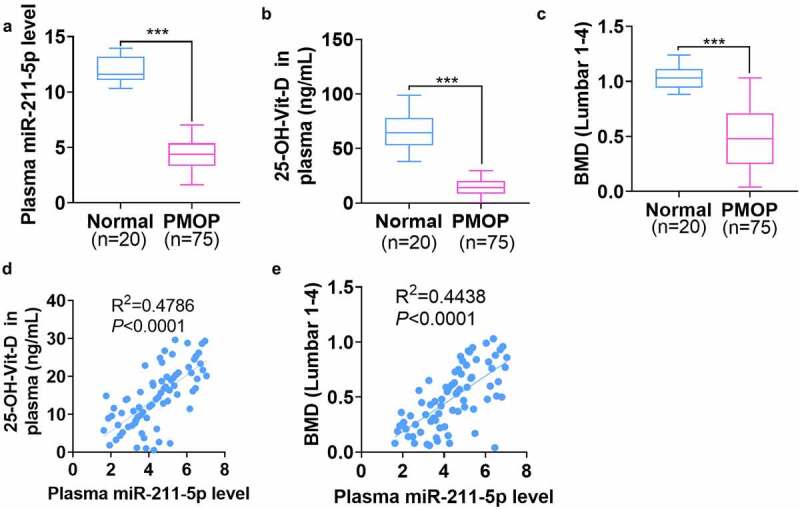
miR-211-5p was knocked down in the plasma of PMOP patients and positively correlated with plasma Vit-D and BMD levels.

The plasma was harvested from 20 healthy controls and 75 PMOP patients. qRT-PCR exhibited that miR-211-5p was knocked down in the plasma of PMOP patients (*P* < 0.001, Figure 1a). The enzyme immunoassay displayed that 25-OH-D concentration was notably attenuated in the plasma of PMOP patients as opposed to that of the healthy controls (*P* < 0.001, Figure 1b). Besides, the lumbar BMD of PMOP patients was evidently lower than that of the healthy patients (*P* < *0.001*, Figure 1c). Next, Pearson analysis revealed that miR-211-5p’s level was positively associated with both the 25-OH-D level (R^2^ = 0.4786, *P* < 0.0001, Figure 1d) and the lumbar BMD level (R^2^ = 0.4438, *P* < 0.0001, Figure 1e) in the patients’ plasma. All these discoveries suggested that miR-211-5p, down-regulated in PMOP patients’ plasma, was positively correlated with Vit-D and BMD levels in the plasma.

The plasma samples were harvested from 20 healthy controls and 75 PMOP patients. (a) qRT-PCR checked miR-211-5p’s expression in the plasma. (b) ELISA determined 25-OH-D concentration in the plasma. (c) Lumbar BMD. (d) The correlation between miR-211-5p and 25-OH-D. (e) The association between miR-211-5p and lumbar BMD. ***P < 0.001 (vs. the Normal group).

### 3.2 miR-211-5p overexpression enhanced hMSCs’ osteogenic differentiation and abated their adipogenic differentiation

**Figure 2.**
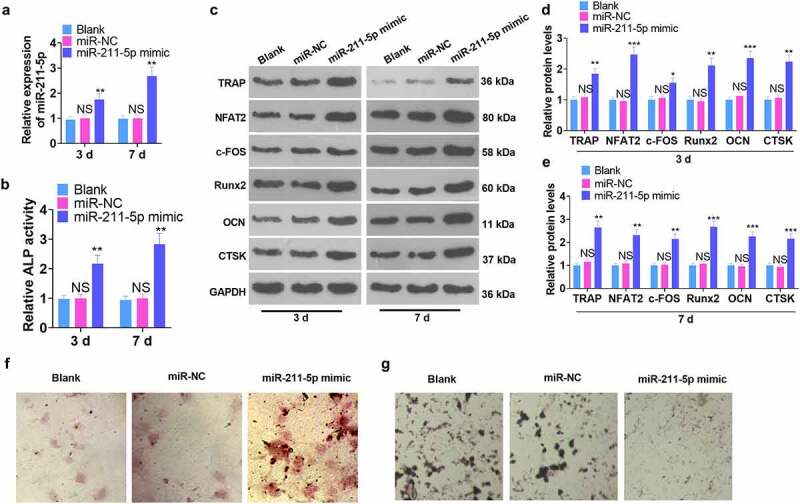
miR-211-5p overexpression enhanced hMSC osteogenic differentiation.

A miR-211-5p overexpression model was engineered in hMSCs. qRT-PCR demonstrated that miR-211-5p’s profile was dramatically higher than that of the miR-NC group on the 3rd and 7th day following the miR-211-5p mimic transfection (*P* < 0.05, Figure 2a). The ALP assay illustrated that miR-211-5p overexpression obviously uplifted ALP activity (vs. the miR-NC group) (*P* < 0.05, Figure 2b). WB confirmed protein expression in hMSCs, suggesting that osteogenic specific proteins (TRAP, NFAT2, c-FOS, Runx2, OCN, and CTSK) were all up-regulated on the 3rd and 7th days subsequent to miR-211-5p overexpression (*P* < 0.05, Figure 2c-e). Alizarin red and oil red O staining further examined the function of miR-211-5p in hMSC osteogenic and adipogenic differentiation. The findings reflected that overexpression of miR-211-5p boosted mineralized matrix formation and lessened lipid droplets in hMSCs (figure 2f-g). These discoveries signified that miR-211-5p overexpression bolstered the osteogenic differentiation and attenuated the adipogenic differentiation of hMSCs.

A miR-211-5p overexpression model was engineered in hMSCs. (a) qRT-PCR confirmed miR-211-5p expression following cell transfection. (b) ALP activity was gauged employing the ALP assay kit. (c-e) WB evaluated the profiles of TRAP, NFAT2, c-FOS, Runx2, OCN, and CTSK. (f-g) Alizarin red staining monitored osteogenic differentiation, while oil red O staining tracked adipogenesis. *P < 0.05, **P < 0.01 (vs. the miR-NC group). N = 3.

### 3.3 miR-211-5p down-regulation impeded the osteogenic differentiation and stepped up the adipogenic differentiation of hMSCs

**Figure 3.**
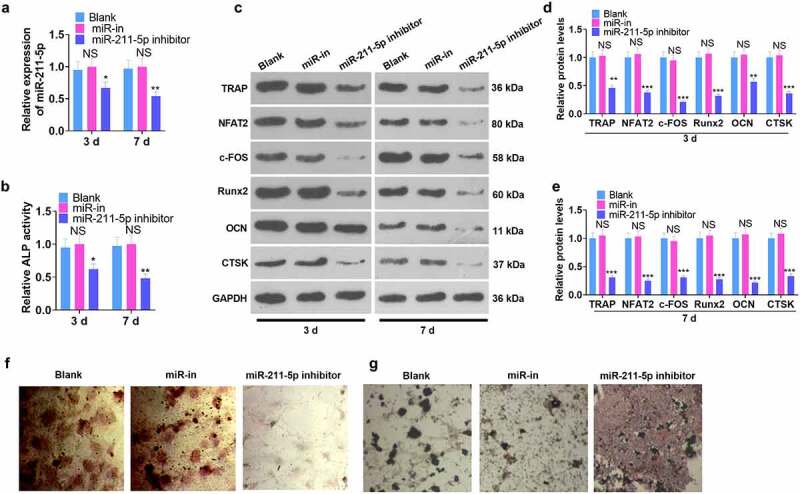
miR-211-5p knockdown hampered hMSC osteogenic differentiation.

hMSCs were transfected along with the miR-211-5p inhibitor and miR-NC, and all the relevant experimental indicators were detected on the 3rd and 7th day after miR-211-5p knockdown. qRT-PCR examined the validity of the transfection. In contrast with the miR-NC group, the miR-211-5p inhibitor evidently suppressed miR-211-5p expression (*P* < 0.05, Figure 3a). ELISA unveiled that miR-211-5p inhibition weakened ALP activity in hMSCs (*P* < 0.05, Figure 3b). WB confirmed that miR-211-5p knockdown hampered the protein profiles of TRAP, NFAT2, c-FOS, Runx2, OCN, and CTSK (*P* < 0.05, Figure 3c-e). Additionally, alizarin red and oil red O staining confirmed that miR-211-5p knockdown abated mineralized matrix formation and augmented lipid droplets in hMSCs (figure 3f-g). In conclusion, miR-211-5p knockdown exerted an inhibitory impact on hMSC osteogenic differentiation.

A miR-211-5p knockdown model was set up in hMSCs. (a) RT-PCR ascertained miR-211-5p profile. (b) The ALP kit was taken to check ALP activity. (c-e) The profiles of TRAP, NFAT2, c-FOS, Runx2, OCN, and CTSK. (f-g) Alizarin red staining examined osteogenic differentiation, whereas oil red O staining checked adipogenesis. *P < 0.05, **P < 0.01 (vs. the miR-NC group). N = 3.

### 3.4 DUSP6 was targeted by miR-211-5p

**Figure 4.**
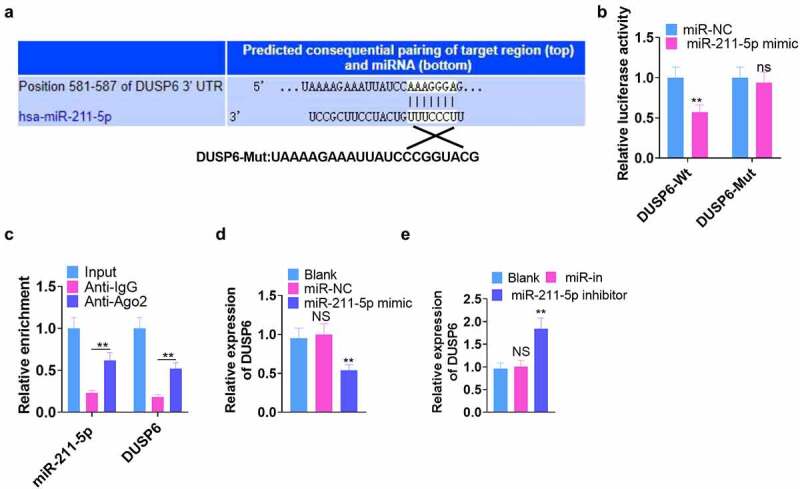
DUSP6 was targeted by miR-211-5p.

We browsed Targetscan (http://www.targetscan.org/) to further investigate the mechanism by which miR-211-5p took effect in hMSCs and discovered that DUSP6 was the binding site of miR-211-5p (Figure 4a). Next, dual-luciferase reporter gene assay verified the binding correlation between the two. As displayed in Figure 4b, miR-211-5p overexpression abated the luciferase activity of the DUSP6-Mt group (*P* < 0.05) but exerted little impact on that of DUSP6-Mut (Figure 4b). RIP analysis disclosed that the Ago2 antibody strengthened miR-211-5p and DUSP6 enrichment (vs. the IgG group) (*P* < 0.05, Figure 4c). qRT-PCR determined DUSP6’s expression, reflecting that miR-211-5p overexpression repressed its expression, whereas miR-211-5p knockdown culminated in the opposite phenomenon (*P* < 0.05, Figure 4d-e). These findings unraveled that DUSP6 was targeted by miR-211-5p.

(a)The base complementary sequences of DUSP6 and miR-211-5p. (b) Dual-luciferase reporter assay assessed the binding correlation between miR-211-5p and DUSP6. ns*P* >0.05, **P < 0.01 (vs. the miR-NC group). (c) RIP analysis verified the association between miR-211-5p and DUSP6. **P < 0.01 (vs. the IgG group). (d) qRT-PCR determined the profile of DUSP6 following miR-211-5p overexpression. ** *P* < 0.01 (vs. the miR-NC group). (e) qRT-PCR revealed DUSP6 expression after miR-211-5p was knocked down. **P < 0.01 (vs. the miR-in group). N = 3.

### 3.5 Overexpression of DUSP6 attenuated the osteogenic differentiation of hMSCs

**Figure 5.**
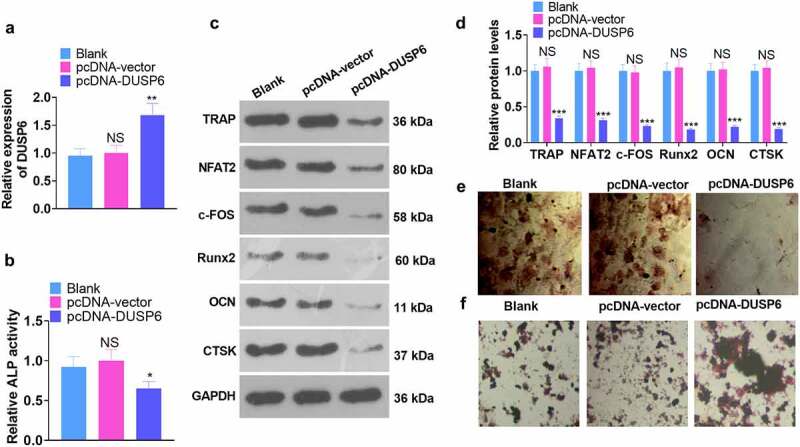
DUSP6 overexpression dampened the osteogenic differentiation of hMSCs.

We engineered a DUSP6 overexpression model to probe the function of DUSP6 in hMSCs’ osteogenic differentiation. All the relevant experimental indicators were examined on the 7th day following cell transfection. qRT-PCR checked the cell transfection efficiency. DUSP6 was conspicuously up-regulated in the pcDNA-DUSP6 group as opposed to the pcDNA-vector group (*P* < 0.05, Figure 5a). As exhibited in Figure 5b, DUSP6 overexpression dampened ALP activity (*P* < 0.05). WB disclosed that overexpression of DUSP6 lowered the profiles of osteogenic specific proteins (TRAP, NFAT2, c-FOS, Runx2, OCN, and CTSK) (vs. the pcDNA-vector group) (*P* < 0.05, Figure 5c-d). Alizarin red and oil red O staining tracked matrix mineralization and lipid droplet formation in hMSCs, signaling that overexpression of DUSP6 frustrated matrix mineralization generation (Figure 5e) and augmented the number of lipid droplets in hMSCs (figure 5f). Thus, it could be concluded that overexpression of DUSP6 impaired hMSCs’ osteogenic differentiation.

A DUSP6 overexpression model was engineered. (a) The profile of DUSP6. (b) The activity of ALP. (c) The profiles of TRAP, NFAT2, c-FOS, Runx2, OCN, and CTSK. (d-e) Alizarin red staining examined osteogenic differentiation, whereas oil red O staining checked adipogenesis. *P < 0.05, **P < 0.01 (vs. the pcDNA-vector group). N = 3.

### 3.6 miR-211-5p overexpression attenuated DUSP6-mediated hMSC osteogenic differentiation inhibition

**Figure 6.**
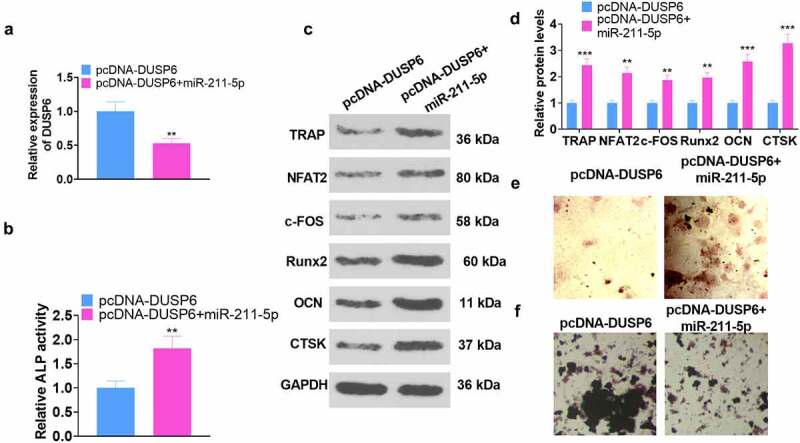
miR-211-5p overexpression abated the DUSP6-mediated inhibition of hMSC osteogenic differentiation.

To confirm the interplay between miR-211-5p and DUSP6, we adopted miR-211-5p mimics to transfect hMSCs already transfected along with pcDNA-DUSP6. All the relevant experimental indicators were tested on the 7th day following the cell transfection. qRT-PCR displayed that DUSP6’s expression was restrained in the pcDNA-DUSP6+ miR-211-5p group (vs. the pcDNA-DUSP6 group) (*P* < 0.05, Figure 6a). As exhibited in Figure 5b, in contrast with the pcDNA-DUSP6 group, overexpression of miR-211-5p vigorously boosted ALP activity (*P* < 0.05). WB unveiled that miR-211-5p overexpression based on DUSP6 overexpression heightened the profiles of TRAP, NFAT2, c-FOS, Runx2, OCN, and CTSK (*P* < 0.05, Figure 6c-d). The images of alizarin red and oil red O staining signaled that as compared with the pcDNA-DUSP6 group, the PCDNA-DUSP6+ miR-211-5p group witnessed a notable rise in matrix mineralization formation (Figure 6e) and a reduction in lipid droplets (figure 6f). In brief, miR-211-5p lowered DUSP6’s expression, hence weakening the DUSP6-mediated inhibition of hMSC osteogenic differentiation.

On the basis of pcDNA-DUSP6 transfection, hMSCs were transfected along with miR-211-5p mimics. (a) DUSP6 expression was lowered. (b) ALP activity. (c) The profiles of TRAP, NFAT2, c-FOS, Runx2, OCN, and CTSK. (d-e) Alizarin red staining examined osteogenic differentiation, whereas oil red O staining tracked adipogenesis. *P < 0.05, **P < 0.01 (vs. the pcDNA-DUSP6 group). N = 3.

### 3.7 Modulation of the ERK-SMAD/β-catenin pathway by miR-211-5p and DUSP6

**Figure 7.**
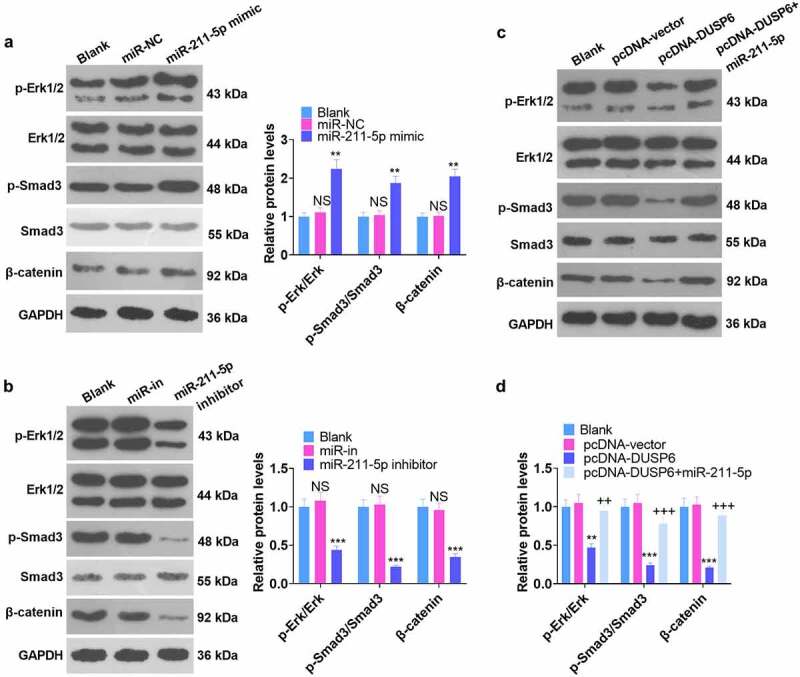
Regulation of the ERK-Smad/β-catenin pathway by miR-211-5p and DUSP6.

Corresponding experimental indicators were examined on Day 7 subsequent to cell transfection. The influence of miR-211-5p and DUSP6 on the ERK-Smad/β-catenin pathway was investigated by WB. As displayed in Figure 7a -B, by contrast to the miR-NC group, miR-211-5p overexpression elevated the levels of p-ERK1/ERK1, p-Smad3/Smad3, and β-catenin, whereas miR-211-5p inhibition completely inverted this situation (*P* < 0.05). Figure 7c-d disclosed that the levels of p-ERK1/ERK1, p-Smad/Smad, and β-catenin were hampered in the pcDNA-DUSP6 group as compared with the pcDNA-vector group, but overexpression of miR-211-5p on this basis impaired such a function of DUSP6 (*P* < 0.05). These phenomena demonstrated that DUSP6 curbed the ERK-SMAD/β-catenin pathway, whereas miR-211-5p boosted the pathway.

(a) WB verified ERK-Smad/β-catenin pathway expression after miR-211-5p overexpression. *P < 0.05 (vs. the miR-NC group). (b) The profile of the ERK-Smad/β-catenin pathway following miR-211-5p knockdown. *P < 0.05, **P < 0.01 (vs. the miR-in group). (c-d) miR-211-5p was overexpressed on the basis of DUSP6, and the profile of the ERK-Smad/β-catenin pathway was ascertained. **P < 0.01 (vs. the pcDNA-vector group). *P < 0.05, **P < 0.01 (vs. the pcDNA-DUSP6 group). N = 3.

### 3.8 Activating the ERK pathway attenuated the DUSP6-mediated inhibition of hMSC osteogenic differentiation

**Figure 8.**
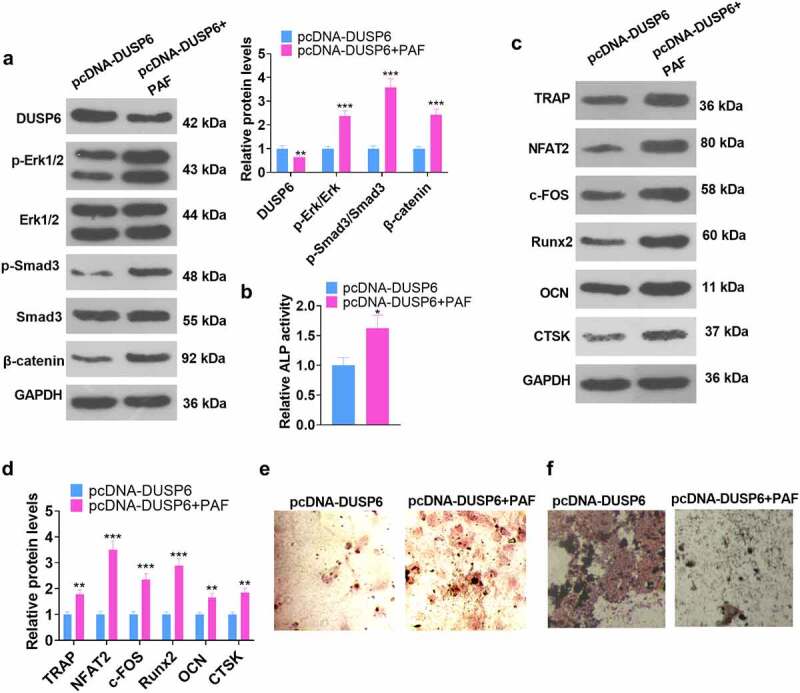
ERK pathway activation impeded the DUSP6-mediated inhibition of hMSC osteogenic differentiation.

To better understand the functions of ERK on DUSP6 in hMSCs, we adopted the ERK agonist PAF-C16 to treat the cells following pcDNA-DUSP6 transfection. Relevant experimental indicators were determined on Day 7 subsequent to the cell transfection. WB unraveled that by contrast to the pcDNA-DUSP6 group, the profile of DUSP6 was lowered, whereas the levels of p-ERK1/ ERK1, p-Smad/Smad, and β-catenin were uplifted (*P* < 0.05, Figure 8a), and those of TRAP, NFAT2, c-FOS, Runx2, OCN, and CTSK were heightened in the pcDNA-DUSP6+ PAF group (*P* < 0.05, Figure 8c-d). ELISA checked ALP activity. As displayed in Figure 8b, as compared with the pcDNA-DUSP6 group, PAF boosted ALP activity (*P* < 0.05). Alizarin red and oil red O staining images exhibited that ERK pathway activation by PAF culminated in increased matrix mineralization formation and lessened lipid droplets generation in hMSCs (Figure 8e-f). These findings unveiled that ERK pathway activation weakened the inhibitory impact of DUSP6 on the osteoblastic differentiation of hMSCs.

Following pcDNA-DUSP6 transfection, hMSCs were dealt with the ERK Agonist PAR-C16 (4 μM). (a) The profiles of DUSP6 and the ERK-Smad/β-catenin pathway. (b) ALP activity. (c-d) The profiles of TRAP, NFAT2, c-FOS, Runx2, OCN, and CTSK. (e-f) Alizarin red staining examined osteogenic differentiation, whereas oil red O staining tracked adipogenesis. *P < 0.05, **P < 0.01 (vs. the pcDNA-DUSP6 group). N = 3.

## 4. Discussion

**Figure 9.**
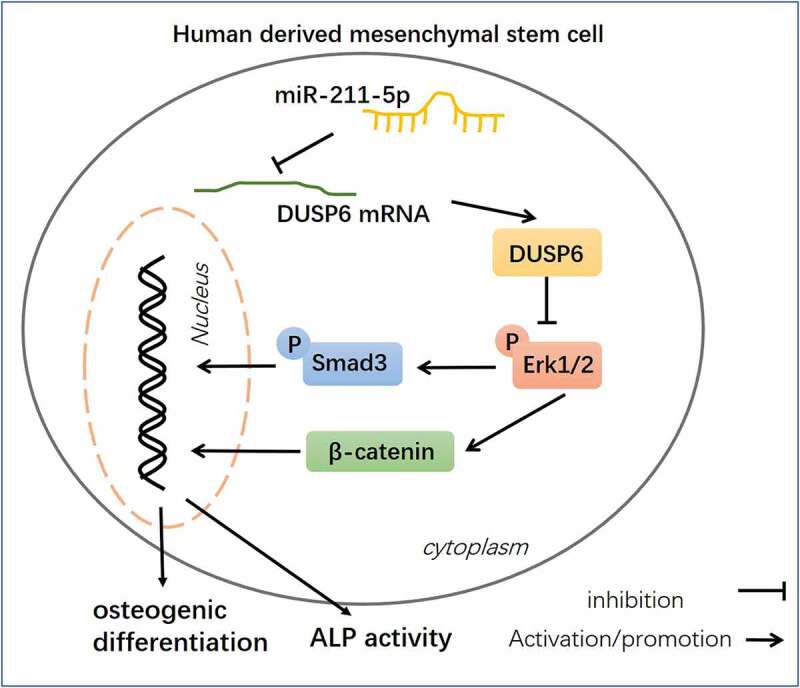
Schematic diagram of miR-211-5p in hMSC osteogenic differentiation.

OP is characterized as the most prevalent chronic metabolic bone disease, and reduced osteogenic differentiation is a significant feature of PMOP [28–30]. Over the years, several studies have verified the correlation between miRNAs and PMOP [31–33]. Here, we discovered that miR-211-5p, down-regulated in the plasma of PMOP patients, targeted DUSP6 to modulate ERK-Smad/β-catenin, hence influencing hMSC osteogenic differentiation (Figure 9).

miR-211-5p targets DUSP6 and inhibits its expression. DUSP6 inactivates the ERK-Smad/β-catenin pathway. MiR-211-5p overexpression promotes ERK-Smad/β-catenin pathway by targeting DUSP6, thus enhancing the osteogenic differentiation of hMSC.

As a member of miRNAs, miR-211-5p has been confirmed with multiple biological functions [34]. During the progression of tumors, miR-211-5p is usually downregulated and exerts a tumor-suppressive role by mediating migration, proliferation and apoptosis [35]. MiR-211-5p mitigates inflammation, apoptosis and cell viability inhibition after cerebral ischemia-reperfusion injury [36]. In terms of bone-related diseases, miR-211-5p also makes a role. For instance, miR-211-5p has a lower level in articular cartilage tissues of osteoarthritis (OA) rat model and enhances chondrocyte differentiation by downregulation Fibulin-4 of ATDC5 cells [37]. In another study, lncRNA PRNCR1 up-regulates CXCR4 by hampering miR-211-5p, thereby repressing osteoblast differentiation and leading to osteolysis following hip replacement [38]. Supported by those studies, we supposed that miR-211-5p can alos affect OP. We examined miR-211-5p’s expression in the plasma samples of 75 PMOP patients and 20 healthy controls. As a result, miR-211-5p was declined in the PMOP group as opposed to the control group. Vit-D is a pivotal factor in OP prevention, and menopause expedites bone loss [39]. Pearson analysis outcomes, aligned with prior works, unveiled that miR-211-5p was positively associated with Vit-D and lumbar BMD levels in the plasma of PMOP patients. Highly efficient osteogenic differentiation of MSCs is critical in treating bone defects and bone diseases [40]. Furthermore, we explored the effects of miR-211-5p on hMSCs differentiation. Our data disclosed that miR-211-5p overexpression expanded the osteogenic differentiation of hMSCs. Nonetheless, knockdown of miR-211-5p gave rise to the opposite effect. Hence, we posit that miR-211-5p up-regulation is conducive to enhance osteogenic differentiation.

DUSP family members, which contain a class of non-receptor-type protein-tyrosine phosphatases, have been found with potent role in mediating osteogenic differentiation [41,42]. For example, METTL3 significantly facilitated osteogenesis ability of BMSCs by mediating the m^6^A modification of DUSP14 [43]. In addition to the marked effects on mediating inflammation and oxidative stress [44,45], DUSP6 has also gained a function on regulation cell differentiation [46–48]. Interestingly, a recent study revealed that miR-181a promotes differentiation and inhibits apoptosis of osteoclasts by directly targeting DUSP6 [49]. Here, TargetScan, dual-luciferase reporter assay, and RIP assay confirmed that miR-211-5p targeted DUSP6 and negatively modulated its profile in hMSCs. Here, we uncovered that overexpression of DUSP6 attenuated hMSCs’ osteogenic differentiation. Moreover, overexpression of miR-211-5p weakened the inhibitory impact of DUSP6 on osteogenic differentiation, which further substantiated the negative regulatory influence of miR-211-5p on DUSP6.

A review of preceding works has enabled us to realize that ERK [50], Smad [51], and β-catenin [52] are crucial in osteogenic differentiation and that activating the ERK, Smad, and β-catenin pathways favors osteogenic differentiation. Here, WB demonstrated that overexpression of miR-211-5p initiated the ERK-Smad/β-catenin pathway. Besides, overexpression of miR-211-5p impaired the inhibitory impact of DUSP6 on ERK-Smad/β-catenin. Further works displayed that ERK pathway activation hindered the DUSP6-mediated inhibition of hMSC osteogenic differentiation. This is consistent with prior studies.

## 5. Conclusion

To summarize, this research unveils miR-211-5p can forecast PMOP, and further mechanism studies display that miR-211-5p curbs DUSP6 to initiate the ERK-Smad/β-catenin pathway, hence boosting hMSC osteogenic differentiation (Figure 9). Nevertheless, other molecular mechanisms that modulate osteogenic differentiation need to be further investigated.

## Data Availability

The data sets used and analyzed during the current study are available from the corresponding author on reasonable request.

## Abbreviations

ALP: alkaline phosphatase
BMD: bone mineral density
DUSP6: dual specific phosphatase 6
hMSCs: human-derived mesenchymal stem cells
LPS: lipopolysaccharide; miRNA: microRNA
miR-in: microRNA inhibitor negative control
MPNST: malignant peripheral nerve sheath tumor
NC: negative control
NS: no significance
OA: osteoarthritis
OP: Osteoporosis
PMOD: Postmenopausal osteoporosis
qRT-PCR: quantitative real-time polymerase chain reaction
SMAD3: SMAD family member 3
Vit-D: vitamin D
